# United States

**Published:** 1898-05

**Authors:** 


### COMMENCEMENT EXERCISES OF THE UNITED STATES COLLEGE OF VETERINARY SURGEONS.

The closing exercises of this school took place Thursday afternoon, April 14, 1898, at 4 o'clock, in the lecture hall of the college, and were witnessed by a large audience. Dr. C. Barnwell Robinson, dean of the faculty, opened the exercises with an appropriate address, and granted the degrees of D.V.S. to the following graduates: M. S. Lantz, of Pennsylvania; G. W. Bready, of Maryland; Theo. T. B. Kirk, of Pennsylvania, and Samuel Gelston and B. W. Gheen, of the District of Columbia.

The degree of fellowship was conferred upon W. A. Hedrick and George A. Prevost, after which Prof. Hedrick addressed the graduates.

Mr. S. Gelston, representing the class of '98, delivered the valedictory address amid great applause. Prof. Prevost then addressed the graduates and friends of the college.

The exercises were closed by remarks from the dean, in which he congratulated the graduates and thanked the friends and patrons for their kind attention and for the many valuable donations received during the session.

